# Performance and Validation of a New Long-Term Mortality Risk Score in Community-Acquired Pneumonia: A Colombian Cohort

**DOI:** 10.3390/idr18050103

**Published:** 2026-09-15

**Authors:** Gabriela Guerron-Gomez, Eduardo Tuta-Quintero, Alirio Bastidas, Luis F. Giraldo-Cadavid, Maria Pérez-Escobar, Luisa F. Martínez, Maria Castillo-Páez, Isabella Criado-Quintero, Manuela Trujillo-Herrera, Angie Sandoval-Blanco, Gabriela Osorio-Betancourt, Laura Medellín-Ortiz, Laura Chaves-Pauwels, Paola Martínez-Sáenz, Luis F. Reyes

**Affiliations:** 1 Department of Epidemiology, Universidad de La Sabana, Campus del Puente del Común, Km. 7, Autopista Norte de Bogotá, Chía 250001, Colombia; gabrielagugo@unisabana.edu.co (G.G.-G.); eduardotuqu@unisabana.edu.co (E.T.-Q.); luisf.giraldo@unisabana.edu.co (L.F.G.-C.); 2 School of Medicine, Universidad de La Sabana, Campus del Puente del Común, Km. 7, Autopista Norte de Bogotá, Chía 250001, Colombia; mariaperes@unisabana.edu.co (M.P.-E.); luisamaga@unisabana.edu.co (L.F.M.); angiesabl@unisabana.edu.co (A.S.-B.);; 3 Critical Care Department, Clínica Universidad de La Sabana, Chía 250001, Colombia; luis.reyes5@unisabana.edu.co; 4 ISARIC, Pandemic Sciences Institute, University of Oxford, Oxford OX1 2JD, UK; 5 Unisabana Center for Translational Science, School of Medicine, Universidad de La Sabana, Chía 250001, Colombia

**Keywords:** community-acquired pneumonia, observational study, performance, mortality

## Abstract

Background: Community-acquired pneumonia (CAP) causes significant long-term morbidity and mortality. Existing clinical scores focus on short-term outcomes, highlighting the need to validate tools that accurately predict 12-month mortality in hospitalized patients. Materials and Methods: A retrospective cohort of adults hospitalized with CAP from 2012 to 2020 was analyzed. Clinical, laboratory, radiological, and hospitalization-related data were collected. A Cox proportional hazards model was developed to predict post-acute mortality between 30 days and 12 months after hospital admission among patients with CAP who survived the first 30 days, with the cohort split 50:50. Model performance was evaluated using Area Under the Receiver Operating Characteristic Curve (AUROC) and standard diagnostic accuracy metrics. Results: A total of 13,851 patients with CAP were included. In the derivation cohort, independent predictors were altered mental status (HR 2.08; 95% CI 1.49–2.90; *p* < 0.05), elevated BUN (>30 mg/dL; HR 1.98; 95% CI 1.48–2.63; *p* < 0.05), temperature extremes (<35 °C or >39.9 °C; HR 1.98; 95% CI 1.43–2.74; *p* < 0.05), corticosteroid use (HR 1.85; 95% CI 1.40–2.44; *p* < 0.05), neoplasia (HR 1.58; 95% CI 1.08–2.32; *p* < 0.05), and hospital stays longer than 8 days (HR 1.39; 95% CI 1.06–1.82; *p* < 0.05). The new score achieved the highest AUROC (0.69; 95% CI: 0.66–0.73), followed by PSI (0.65; 95% CI: 0.62–0.69), CURB-65 (0.63; 95% CI: 0.59–0.67), and CAPSI (0.62; 95% CI: 0.58–0.67). The optimal cutoff point for the new score was 3, as determined by the Youden index (0.339). The model sensitivity was 83.0%, specificity: 50.9%, PPV: 8.9%, and NPV: 99.0%. The LR+ was 1.69 (95% CI: 1.38–2.06), and the LR− was 0.33 (95% CI: 0.27–0.41). Conclusions: The new score demonstrated weak-to-moderate discriminatory capacity. The variables included in the new score reflect multiorgan involvement, disease severity, and comorbidity burden, all of which are associated with long-term mortality.

## 1. Introduction

Community-acquired pneumonia (CAP) is a major cause of global morbidity and mortality. Although clinical scores such as PSI and CURB-65 are widely used to predict 30-day mortality with good accuracy [1,2], their ability to estimate long-term outcomes, including 12-month mortality, remains limited [1,3]. Data from the Centers for Disease Control and Prevention (CDC) similarly show that 16% of CAP patients die between days 31 and 365 after discharge, with mortality increasing markedly with age [4].

Beyond mortality, CAP has been associated with a higher risk of complications such as stroke. Wang et al. found that patients with bacterial pneumonia had a significantly increased likelihood of ischemic (HR: 5.72) and hemorrhagic stroke (HR: 5.33), underscoring the systemic effects of pneumonia and the need for long-term follow-up [5]. Cardiovascular mortality is also increasingly reported among CAP survivors, rising from 16% during hospitalization to 24% at one year post-discharge [4]. These findings highlight the importance of identifying patients at elevated risk of adverse outcomes long after initial recovery.

Various clinical scores have been developed to stratify CAP severity. While PSI and CURB-65 remain the most validated tools for predicting 30-day mortality [6,7,8], other scores designed for short-term risk estimation include NEWS, CORB, SCAP, REA-ICU, PNEUMOSHOCK, and A-DROP [7,9,10,11,12,13,14]. However, most of these instruments lack validation for long-term mortality. This gap has prompted efforts to develop tools specifically aimed at predicting 12-month outcomes, such as the CAPSI (Community-Acquired Pneumonia Severity Index) and UBMo (uremia, N-terminal pro-brain natriuretic peptide, plasmatic rate, monocyte count) [14,15].

Despite these advances, current available tools remain primarily focused on short-term outcomes, and those proposed for long-term use demonstrate only weak-to-moderate discriminatory capacity, leaving a critical gap in extended risk stratification. In Colombia, local studies evaluating existing scores for 30-day mortality have yielded only moderate performance [16,17]. Consequently, there is a clear need for clinical risk stratification tools capable of identifying patients at increased risk of post-acute mortality following hospitalization for CAP. Therefore, this study aimed to develop and internally validate a clinical risk score for predicting mortality between 30 days and 12 months after hospital admission among patients with community-acquired pneumonia who survived the first 30 days of hospitalization at a tertiary-level hospital in Colombia. 

## 2. Materials and Methods

A retrospective cohort study was conducted including adult patients admitted to the Emergency Department, general wards, or ICU at Clínica Universidad de La Sabana in Chía, Colombia, between 2012 and 2020 with a diagnosis of CAP. The study was conducted at Clínica Universidad de La Sabana, a tertiary care academic hospital in Chía, Colombia, affiliated with the Universidad de La Sabana. The institution provides 24/7 emergency care and comprehensive adult and pediatric services. At the time of the study, it had approximately 189 inpatient beds, including isolation rooms, and dedicated adult intensive and intermediate care units. This was a retrospective study, and informed consent was not required. The reporting of this study conforms to STROBE guidelines [18].

### 2.1. Inclusion Criteria

Patients aged 18 years or older were included if they met the diagnostic criteria for CAP according to the Infectious Diseases Society of America/American Thoracic Society (IDSA/ATS) guidelines: clinical symptoms of acute respiratory infection accompanied by radiographic findings consistent with alveolar and/or interstitial infiltrates [19]. Patients with incomplete clinical records that prevented calculation of any of the risk scores were excluded.

To specifically evaluate mortality beyond the acute phase of illness, 30-day mortality was used as the threshold for acute mortality, consistent with previous prognostic and epidemiological studies of hospitalized patients with CAP, in which 30-day mortality is a commonly used outcome measure [1,8]. Patients who died within the first 30 days of hospital admission were therefore excluded from the final analytic cohort. The time-to-event analysis included only patients who survived at least 30 days, with follow-up extending from day 30 after admission to 12 months. This approach was chosen to specifically assess post-acute mortality risk beyond the commonly evaluated 30-day period.

### 2.2. Data Collection

Data was obtained from electronic medical records and included sociodemographic information, clinical variables, physiological parameters, and comorbidities, all collected at hospital admission, with comorbidity burden assessed using the Charlson Comorbidity Index. Laboratory values obtained at admission included complete blood count, blood urea nitrogen, glucose, serum albumin, bilirubin, and arterial blood gases. Chest X-rays performed at admission were evaluated and interpreted by institutional radiologists. Mortality data were obtained through the national death registry.

Data was manually extracted from the electronic medical records by trained investigators using a standardized data collection form. To ensure data quality and consistency, predefined variable definitions were applied, and records were reviewed systematically at the time of data collection. Any discrepancies were resolved by consensus among the study investigators.

Medical records needed to include sufficient information for the evaluation of CURB-65, CRB-65, Severe Community-Acquired Pneumonia (SCAP), CORB, ADROP, National Early Warning Score (NEWS), Pneumonia Shock, Risk of Early Admission to ICU (REA-ICU), PSI, SMART-COP, SMRT-CO, SOAR, Quick Sequential Organ Failure Assessment (qSOFA), Systemic Inflammatory Response Syndrome (SIRS), CAPSI, and Charlson Comorbidity Index (CCI) scores (Supplementary Tables S1 and S2). The new score integrates clinical, laboratory, comorbidity, and hospitalization-related variables associated with 12-month mortality in patients with CAP who survived the first 30 days after hospital admission. Variables were categorized according to their timing of availability: admission-based variables included altered mental status, multilobar or bilateral infiltrates, BUN > 30 mg/dL, extreme temperature, and age ≥ 70 years, whereas hospital-course variables included neoplasia, chronic kidney disease (CKD), hospital stay > 8 days, and corticosteroid use during hospitalization. The latter variables were incorporated as post-admission prognostic markers and are not intended for use at the time of initial presentation. 

### 2.3. Procedures for Clinical Screening and Data Extraction

Eligibility confirmation required detailed chart review to verify explicit documentation of both compatible respiratory symptoms and radiographic evidence at presentation. To ensure uniform case ascertainment, two physicians with prior experience in pneumonia management conducted the screening process following a structured review protocol. During the screening phase, records were initially assessed by one reviewer, and a proportion was independently re-evaluated by a second reviewer to assess consistency in eligibility determination. Disagreements regarding symptom documentation or eligibility were adjudicated through joint review. This verification process was completed prior to final cohort assembly to reduce the risk of misclassification during patient selection. 

### 2.4. Missing Data

Missing data was assessed for all study variables. Variables with >10% missing data were excluded from the analysis and were not imputed. For variables with <10% missingness, the imputation method was selected according to the nature of the variable (Supplementary Table S3). Quantitative variables were imputed using weighted mean imputation, whereas categorical variables were imputed using regression-based methods. Binary categorical variables were imputed using binary logistic regression, while categorical variables with more than two levels were imputed using multinomial logistic regression [20]. The imputation models incorporated the available clinical and demographic information to estimate the most likely value for each missing observation. To assess the robustness of the findings, analyses using the original non-imputed data were compared with those obtained after imputation, and no substantial differences were observed.

### 2.5. Sample Size

Sample size considerations were based on recommendations for the development and validation of clinical prediction models [21]. For model derivation, at least 10 outcome events per candidate predictor parameter were targeted to minimize overfitting. Given nine candidate predictors, a minimum of 90 deaths at 12 months was required, corresponding to approximately 360–450 patients assuming a 20–25% event rate. For internal validation, a minimum of 50 outcome events was considered adequate to allow reliable estimation of model performance.

### 2.6. Statistical Analysis

Analyses were performed using IBM SPSS Statistics version 25 (IBM Corp., Armonk, NY, USA) and RStudio version 2025.05.1+513 (Posit, PBC, Boston, MA, USA). Continuous variables were described as mean ± SD or median (IQR), and categorical variables as frequencies [20]. Bivariate comparisons used *t*-test, Mann–Whitney U test, or chi-square as appropriate [20]. The study population was randomly divided into two independent cohorts using a 50:50 split: a derivation cohort and an internal validation cohort. All candidate predictors were selected and evaluated exclusively in the derivation cohort, where univariable and multivariable analyses were performed to construct the prognostic model. The internal validation cohort was used solely to evaluate the performance of the final model, including discrimination and calibration, without any re-estimation of coefficients or identification of additional predictors. Variables with *p* < 0.20 in univariable analysis or considered clinically relevant were entered into a multivariable Cox proportional hazards model to identify predictors of post-acute mortality between day 30 and day 365 among 30-day survivors [20]. Time-to-event was calculated from day 30 after hospital admission until death or day 365. The final set of predictors was determined based on their independent association with post-acute mortality, statistical significance, clinical plausibility, and the absence of substantial redundancy between predictors.

The final multivariable Cox model provided the regression coefficients (β) and hazard ratios (HRs) for each independent predictor. These regression coefficients were used to derive the clinical risk score [20]. Each predictor was assigned an integer number of points proportional to its regression coefficient, with predictors having larger absolute coefficients receiving greater weights. The individual points were then summed to obtain a total score for each patient. The resulting score was evaluated in the derivation cohort and subsequently applied without modification to the independent internal validation cohort [20].

The proportional hazards assumption was assessed using scaled Schoenfeld residuals and the Grambsch–Therneau test. The test was performed for each predictor included in the Cox proportional hazards model and for the model globally [20]. A *p* value <0.05 was considered indicative of a statistically significant departure from the proportional hazards assumption [20].

### 2.7. Predictor Variables and Operational Definitions

The variables included in the final prognostic score were selected based on their independent association with post-acute mortality in the multivariable Cox regression model and their clinical relevance [20]. Operational definitions and thresholds were established a priori to ensure consistency and reproducibility. Altered mental status was defined as any documented acute change in consciousness or cognition at presentation. Multilobar or bilateral infiltrates were identified on chest radiography interpreted by the treating physician or radiologist. BUN was considered elevated when >30 mg/dL at admission. Abnormal temperature was defined as hypothermia (<35 °C) or hyperthermia (>39.9 °C) recorded at presentation. Age was categorized as ≥70 years. Cutoffs for age, blood urea nitrogen, and temperature were selected a priori based on established clinical thresholds and prior pneumonia severity scores, aiming to enhance clinical interpretability and facilitate bedside application [9,11,14,16,19].

Neoplasia was defined as any active solid or hematologic malignancy, regardless of treatment status. Kidney disease was defined as a documented diagnosis of CKD prior to admission. Corticosteroid use was defined as systemic corticosteroid therapy administered during hospitalization. Length of hospital stay >8 days was calculated from admission to discharge. The selected variables encompassed markers of acute illness severity, indicators of host vulnerability and comorbidity burden, as well as factors related to treatment-induced immunosuppression.

The derived model assigned points based on regression coefficients, and Youden’s index determined the optimal cutoff [20]. The cohort was randomly split into derivation and internal validation sets in a 50:50 ratio. Model performance (sensitivity, specificity, positive predictive value (PPV), negative predictive value (NPV), and likelihood ratios with 95% CI) was calculated, and Area Under the Receiver Operating Characteristic Curve (AUROC) for the following scores were assessed: CURB-65, CRB-65, SCAP, CORB, A-DROP, NEWS, Pneumonia Shock, REA-ICU, PSI, SMART-COP, SMRT-CO, SOAR, qSOFA, SIRS, CAPSI, and Charlson. AUROCs were classified as follows: 0.50–0.59, very poor discrimination; 0.60–0.69, weak to moderate; 0.70–0.79, acceptable (moderate); 0.80–0.89, good; and 0.90–1.00, excellent discrimination. The AUROC of the new score was compared with PSI, CURB-65, and SMART-COP using DeLong’s test (*p* < 0.05) [20]. 

Model calibration was assessed in the validation cohort by comparing predicted and observed 12-month post-acute mortality. Calibration in the large and the calibration slope were estimated with their corresponding 95% confidence intervals, and the Brier score was calculated as a measure of overall prediction error [20]. Calibration was also examined graphically by plotting observed mortality against mean predicted mortality across prognostic score categories, with the 45-degree line representing perfect calibration [20].

## 3. Results

**Figure 1:**
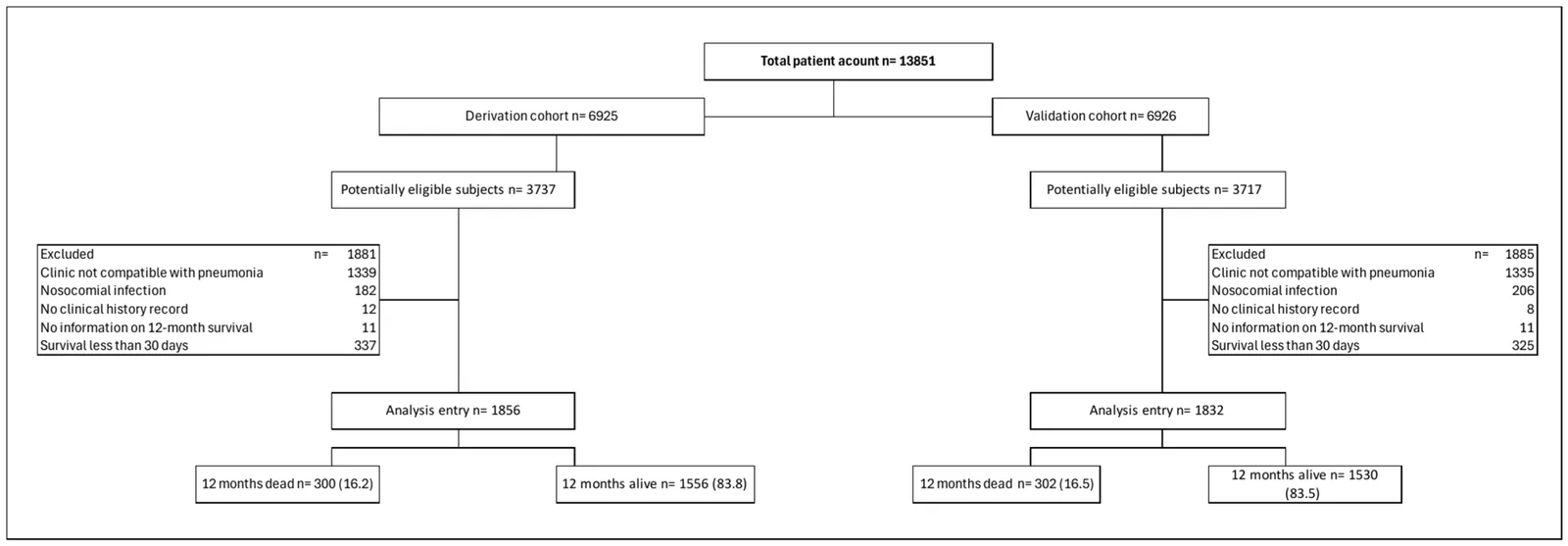
Flowchart for subject enrollment in the study.

A total of 13,851 patients with CAP were initially included. The cohort was randomly divided into two groups: derivation (*n* = 6925) and validation (*n* = 6926). In the derivation cohort 1856 patients entered the analysis while 1832 were included in the validation cohort (Figure 1).

In both cohorts, non-survivors were significantly older than survivors. In the derivation cohort, mean age was higher among patients who died compared with those who survived (69.5 ± 18.1 vs. 62.0 ± 21.8 years, *p* < 0.05), a pattern that was consistent in the validation cohort (71.9 ± 16.9 vs. 62.0 ± 21.8 years, *p* < 0.05). The most frequent symptoms at presentation in the overall cohorts were cough (83.3% in the derivation cohort and 81.9% in the validation cohort) and dyspnea (69.0% and 65.8%, respectively). Hypertension was associated with mortality in the validation cohort (*p* = 0.005), while chronic lung disease was significantly associated with mortality in the derivation cohort (*p* < 0.05). Chronic heart failure was significant only in the validation cohort (*p* < 0.05), whereas tumor was significantly associated with mortality in both cohorts. CKD was more frequent among non-survivors in both cohorts, reaching statistical significance in the validation cohort (10.3% vs. 5.0%, *p* < 0.05), while a similar but non-significant trend was observed in the derivation cohort (7.0% vs. 4.7%, *p* = 0.093) (Table 1).

Among laboratory variables, non-survivors had lower hemoglobin (13.0 vs. 13.7 g/dL, *p* < 0.05), and lower hematocrit (38.5% vs. 40.8%, *p* < 0.05). Electrolyte levels were similar between groups, but serum sodium was slightly higher in non-survivors in the validation cohort (138.3 vs. 136.8 mmol/L, *p* < 0.05). Regarding radiological findings, interstitial infiltrates were more common among non-survivors (58.6% vs. 44.4%, *p* < 0.05), while alveolar infiltrates were slightly more frequent in survivors. Among medical interventions, non-survivors had significantly higher use of vasopressors (13.0% vs. 5.8%, *p* < 0.05), corticosteroids (32.0% vs. 17.9%, *p* < 0.05), invasive mechanical ventilation (10.0% vs. 6.2%, *p* < 0.05), and non-invasive ventilation (5.7% vs. 2.5%, *p* < 0.05). (Supplementary Table S4)

### 3.1. Cox Regression of New Score Included Variables

In the derivation cohort, independent predictors of 12-month mortality included altered mental status (HR 2.08; 95% CI 1.49–2.90; *p* < 0.05), elevated BUN (>30 mg/dL; HR 1.98; 95% CI 1.48–2.63; *p* < 0.05), temperature extremes (<35 °C or >39.9 °C; HR 1.98; 95% CI 1.43–2.74; *p* < 0.05), corticosteroid use (HR 1.85; 95% CI 1.40–2.44; *p* < 0.05), neoplasia (HR 1.58; 95% CI 1.08–2.32; *p* < 0.05), and hospital stays longer than 8 days (HR 1.39; 95% CI 1.06–1.82; *p* < 0.05). Multilobar infiltrates on chest X-ray also remained significant (HR 1.46; 95% CI 1.10–1.93; *p* < 0.05).

The validation cohort confirmed these findings, with significant predictors including altered mental status (HR 1.65; 95% CI 1.20–2.27; *p* < 0.05), BUN > 30 mg/dL (HR 1.37; 95% CI 1.03–1.83; *p* < 0.05), age ≥ 70 years (HR 1.59; 95% CI 1.21–2.10; *p* < 0.05), corticosteroid use (HR 1.57; 95% CI 1.19–2.07; *p* < 0.05), neoplasia (HR 2.34; 95% CI 1.64–3.36; *p* < 0.05), CKD stage ≥ 3 (HR 1.70; 95% CI 1.13–2.53; *p* < 0.05), multilobar infiltrates (HR 1.53; 95% CI 1.16–2.01; *p* < 0.05), and hospital stay > 8 days (HR 1.51; 95% CI 1.15–1.97; *p* < 0.05). Temperature extremes were not significant in validation (*p* = 0.439) (Table 2). The proportional hazards assumption was formally assessed using scaled Schoenfeld residuals and the Grambsch–Therneau test. No evidence of violation of the proportional hazards assumption was identified either for the individual predictors or for the model globally (global χ^2^ = 7.204, df = 9, *p* = 0.616) (Supplementary Table S5).

### 3.2. New Score Performance

When comparing the predictive performance of different severity scores for 12-month mortality in the validation cohort, the new score achieved the highest AUROC (0.69; 95% CI: 0.66–0.73), followed by PSI (0.65; 95% CI: 0.62–0.69), CURB-65 (0.63; 95% CI: 0.59–0.67), and CAPSI (0.62; 95% CI: 0.58–0.67). In contrast, the lowest discriminative performance was observed for SIRS, with an AUROC of 0.48 (95% CI: 0.45–0.52) (Table 3).

The optimal cutoff point for the new score was 3, as determined by the Youden index (0.339). The model sensitivity was 83.0%, specificity 50.9%, PPV 8.9%, and NPV 99.0%. The positive likelihood ratio (LR+) was 1.69 (95% CI: 1.38–2.06), and the negative likelihood ratio (LR−) was 0.33 (95% CI: 0.27–0.41).

**Figure 2:**
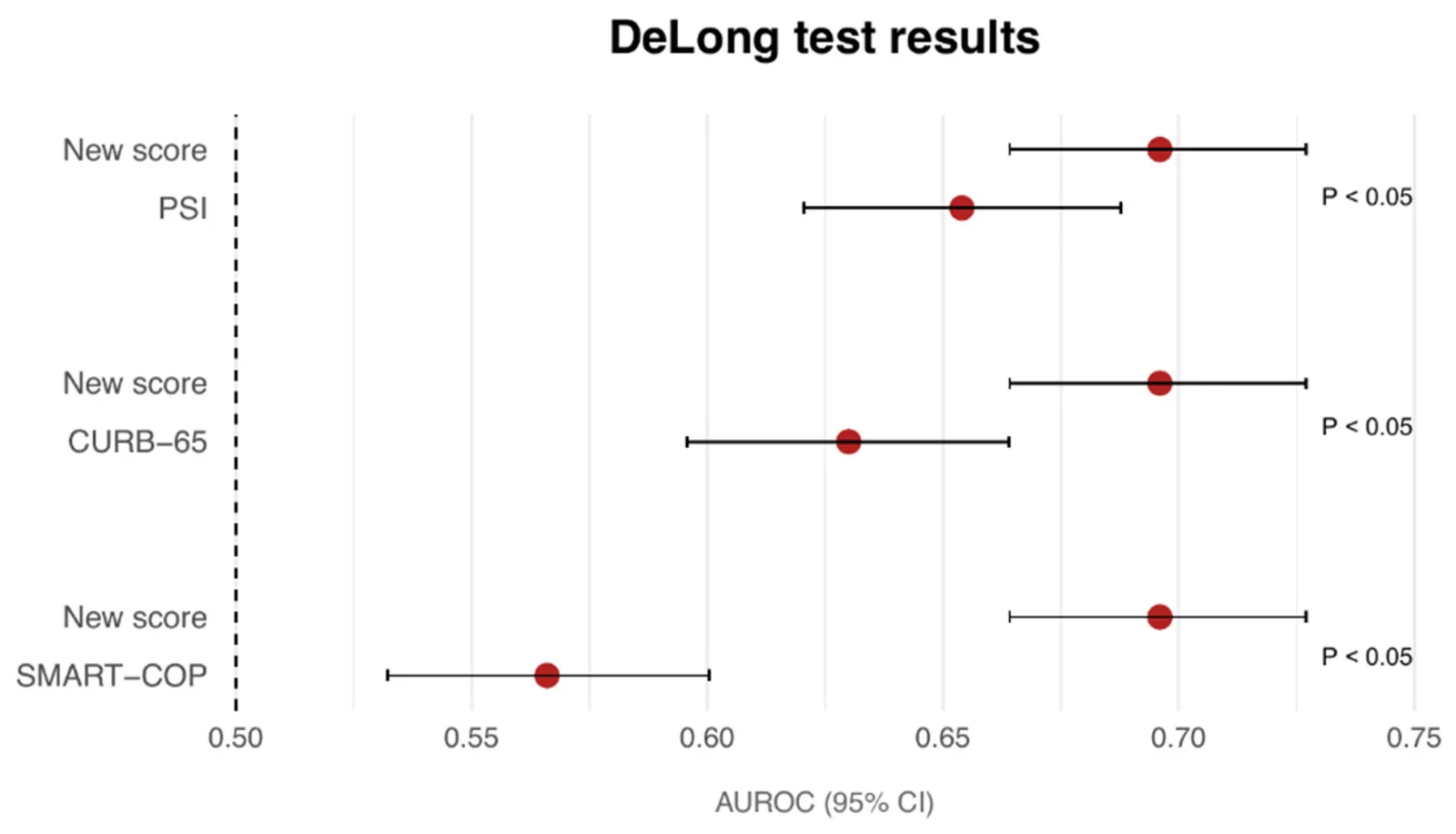
AUROC differences between the new prognostic score and existing clinical scores using the DeLong test. Notes: The new score showed significantly higher AUROC compared to PSI (Δ = 0.057, *p* ≤ 0.05), CURB-65 (Δ = 0.063, *p* ≤ 0.05), and SMART-COP (Δ = 0.127, *p* ≤ 0.05). All comparisons were statistically significant (*p* < 0.05). The dashed line represents the null hypothesis (Δ = 0).

As shown in Figure 2, the new score demonstrated superior discriminative performance compared with PSI (Δ = 0.057; *p* < 0.05), CURB-65 (Δ = 0.063; *p* < 0.05), and SMART-COP (Δ = 0.127; *p* < 0.05), with all comparisons performed using the DeLong test. All performance metrics shown in Table 3 were calculated exclusively in the validation cohort.

**Figure 3:**
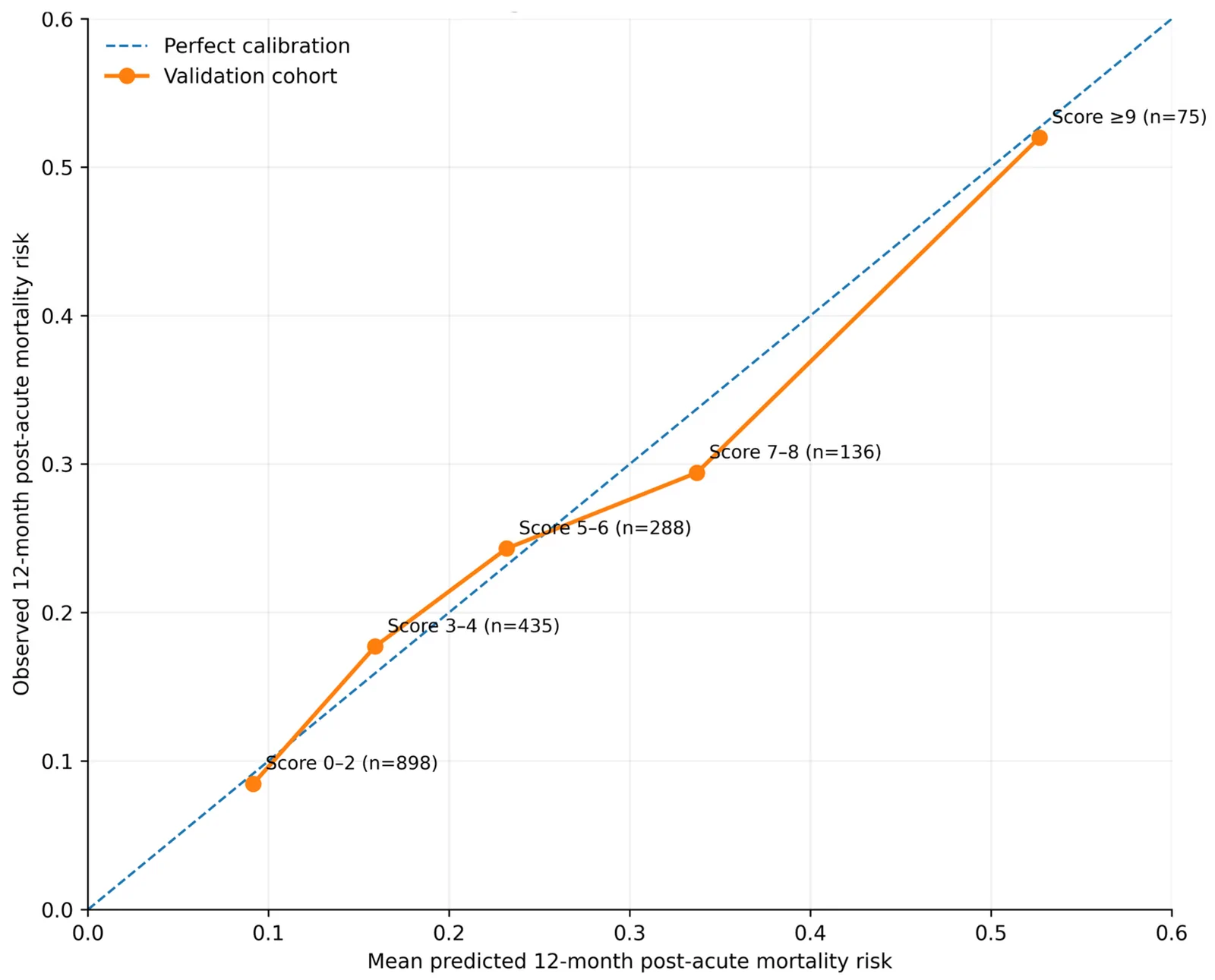
Calibration plot of the prognostic score in the validation cohort. Notes: Observed versus mean predicted 12-month post-acute mortality across prognostic score categories. The diagonal dashed line represents perfect calibration; points closer to the diagonal indicate better agreement between predicted and observed mortality.

The calibration in the large was −0.045 (95% confidence interval, −0.335 to 0.246), and the calibration slope was 0.974 (95% confidence interval, 0.800 to 1.149). The Brier score was 0.128. Mean predicted mortality was 16.6%, compared with an observed mortality of 16.5%. Across increasing score categories, observed and predicted mortality showed close agreement, as illustrated in the calibration plot (Figure 3 and Supplementary Table S6). 

## 4. Discussion

The new score demonstrated weak-to-moderate discriminatory capacity. The variables included in the new score reflect multiorgan involvement, disease severity, and comorbidity burden, all of which are associated with long-term mortality. Patients with multiple comorbidities who required vasopressors or mechanical ventilation experienced lower survival rates, reflecting greater disease severity. While the new score achieved the highest AUROC among the evaluated tools, its overall discriminative performance remained weak to moderate.

Emerging evidence indicates that the impact of CAP extends well beyond the acute phase. Myint et al. reported all-cause mortality rates of 24.3% during the first year (HR: 7.3) and 47.8% beyond one year (HR: 2.8) among hospitalized patients, along with significant increases in respiratory and cardiovascular deaths [3]. In this context, our findings further support the importance of assessing long-term prognosis after hospitalization for CAP [21]. The identification of patients at increased risk of 12-month mortality may be clinically relevant for planning post-discharge follow-up and identifying individuals who may benefit from closer surveillance and management of persistent or underlying health conditions [2,3]. Thus, the proposed score may provide complementary prognostic information beyond tools primarily designed to assess acute CAP severity.

When comparing the performance of the new score with others, such as CAPSI, PSI, and CURB-65, these scores showed higher AUROCs in their original derivation cohorts, although their performance tends to decrease or remain stable in subsequent validation studies. This difference may be explained by the fact that these risk scores primarily rely on acute physiological markers, such as respiratory rate ≥ 30 breaths/min, systolic blood pressure < 90 mmHg, extreme temperatures (<35 °C or >39.9 °C), and altered mental status, which may limit their ability to predict long-term mortality [7,11,12,13,14,22]. In contrast, our model incorporates a broader range of chronic and acute variables; however, its discriminative capacity remains modest and should be interpreted as a complementary tool rather than a definitive predictor of long-term mortality.

In our cohort, many hospitalized patients with CAP had chronic comorbidities, including chronic lung disease, heart failure, and diabetes mellitus. These comorbidities were common, consistent with observations from previous cohorts evaluating risk scores [7,11,12,13,14,22]. These findings suggest that our cohort is representative of the population typically affected by CAP and highlight the importance of considering chronic comorbidities when assessing prognosis and guiding clinical management in this patient group.

The variables included in the new score indicate that patients with multiorgan involvement, severity markers, and comorbidities such as renal failure and neoplasm have a higher risk of 12-month mortality. Among these, altered mental status was the strongest predictor. A prospective cohort study identified altered mental status as an independent predictor of increased medium-term mortality after CAP hospitalization [23]. Additionally, 12-month mortality among CAP patients presenting with altered mental status is higher than in the general CAP population [15,24]. Altered mental status may reflect neurological dysfunction secondary to severe systemic involvement, including hypoxemia or sustained inflammation, but may also serve as a marker of underlying frailty [24,25]. Reduced physiological reserve and increased susceptibility to delirium in frail patients may contribute to long-term deterioration and help explain the strong association with 12-month mortality observed in our cohort [24,25].

Prolonged hospital stay in CAP often reflects greater disease severity, complications, and comorbidities [26,27]. In our cohort, corticosteroids were primarily administered to critically ill patients to modulate the inflammatory response and reduce the risk of ARDS and mechanical ventilation. The higher mortality observed in these patients likely reflects their more severe clinical condition [28]. Additionally, abnormal body temperatures, such as hypothermia and persistent hyperthermia, serve as markers of systemic inflammation and have been associated with poorer outcomes, potentially indicating ongoing infection or long-term immune dysregulation [26,27,28,29].

Neoplasia was an important independent risk factor for mortality in our cohort, highlighting its prognostic and clinical relevance. This may be explained by tumor-associated immunosuppression and other related mechanisms [30,31], which increase susceptibility to infections and contribute to a worse prognosis. Moderate to severe CKD showed a smaller effect in the Cox regression model; however, one-year mortality after hospitalization for pneumonia reaches approximately 45% in patients with end-stage renal disease on hemodialysis [32]. Additionally, the new score included a BUN ≥ 30 mg/dL, which may reflect persistent physiological alterations, such as worsening chronic kidney function or advanced renal failure [7,11]. This finding may be explained by the heterogeneity in the definition of chronic kidney disease in our cohort, which included patients with different stages of renal dysfunction, most of whom were not receiving renal replacement therapy. Future studies should differentiate outcomes between patients with end-stage kidney disease on hemodialysis and analyze more homogeneous populations to allow a more accurate estimation of risk [7,11,32].

Patients requiring mechanical ventilation or vasopressors showed lower 12-month survival, reflecting greater systemic compromise and reduced physiological reserve [33,34,35]. These markers of severe acute illness are clinically relevant and help contextualize the performance of the new score, which incorporates variables reflecting both chronic comorbidity and acute severity to predict long-term mortality [34,35].

From a clinical perspective, the proposed score could serve as a tool for identifying 30-day survivors of CAP who may benefit from intensified post-discharge assessment and targeted follow-up [36,37]. Patients classified as higher risk could potentially be considered for earlier outpatient reassessment, optimization of comorbidities and medications, evaluation of nutritional and functional status, pulmonary or cardiac rehabilitation when appropriate, vaccination and preventive care, and closer monitoring for recurrent respiratory or cardiovascular events [38]. Importantly, the score should not be interpreted as an indication for a specific intervention or as a substitute for clinical judgment. Rather, it may provide a framework for identifying patients who warrant a more comprehensive post-acute care pathway.

### Limitations

Its retrospective design limits control over data quality and introduces potential information bias; however, the large sample size collected over several years enhances generalizability. Although some variables required imputation because of missing data, regression-based and mean-based imputation methods were applied according to the nature of the variables [20]. A substantial proportion of the initial cohort was excluded, mainly because incomplete clinical records prevented calculation of the evaluated risk scores, which may have introduced selection bias and further limits generalizability. In addition, cause-of-death information was not available; therefore, competing risks could not be assessed, and the proposed model predicts overall long-term mortality rather than CAP-specific mortality. Although the new score demonstrated statistically significant discrimination compared with commonly used prognostic scores, its overall predictive performance was modest, which may limit its standalone clinical applicability and underscores the need for external validation in independent populations.

The statistical methods applied were considered appropriate and sufficient for the objectives of this study [20]. However, the use of randomized splitting for derivation and validation, as well as the variable selection strategy based on predefined thresholds and clinical plausibility, may be associated with a risk of overfitting and reduced model stability. These aspects may limit the generalizability of the findings and should be addressed in future studies using alternative validation approaches and penalized regression techniques.

Cause-specific mortality could not be assessed; nonetheless, 12-month all-cause mortality remains a clinically relevant endpoint. The model relies on routinely collected clinical data, supporting its applicability in resource-limited settings. In addition, this study focused primarily on the discriminative ability of the proposed score to stratify long-term mortality risk in hospitalized patients with CAP.

Age, BUN, and temperature were modeled as dichotomous variables using clinically established thresholds. While this approach facilitates clinical interpretation, practical application, and parsimony in the resulting score, dichotomizing continuous variables can lead to a loss of prognostic information, mask potential nonlinear associations, and reduce discriminatory power compared to maintaining continuous measurements [20]. Finally, the proportional hazards assumption of the Cox model was not formally assessed in the original analysis. Consequently, potential violations of this assumption cannot be ruled out, which could affect the estimated hazard ratios and their interpretation during the follow-up period. If the effect of a predictor changes over time, a constant hazard ratio might not adequately represent its prognostic association [20].

An additional limitation of this study is the absence of a formal calibration assessment. While discrimination was the primary focus, the lack of calibration charts or metrics prevents an evaluation of how closely the predicted risks match the observed outcomes across risk strata. Formal calibration analyses were not performed because the score was not intended to estimate absolute individual risk, but rather to support clinical risk stratification [20,39]. Incorporating calibration into future validation studies will be important to fully establish the accuracy and clinical utility of the proposed score.

Early mortality in CAP is primarily driven by acute complications such as septic shock, respiratory failure, and multiorgan dysfunction, whereas mortality beyond 30 days is more strongly associated with persistent systemic inflammation, immune dysregulation, comorbidity burden, and reduced physiological reserve [10,19,27]. Because our objective was to evaluate predictors of long-term vulnerability and the performance of prognostic scores beyond the acute phase, the analysis was restricted to patients surviving the first 30 days. This approach is consistent with our previous work, in which patients with nosocomial infection, incomplete records, missing 12-month survival data, or survival < 30 days were excluded to specifically assess long-term mortality among 30-day survivors hospitalized with CAP [39]. We acknowledge that this conditional survival design may introduce survival conditioning bias and limit generalizability to all patients from the time of admission.

As the analysis was limited to patients who survived the first 30 days, the score is best conceptualized as a post-acute prognostic instrument, intended for use after initial clinical stabilization rather than as an admission-based triage tool [39]. The administration of corticosteroids is likely to reflect a higher inflammatory burden or clinical deterioration during hospitalization and may therefore be influenced by confounding by indication [40,41]. Although malignancy may increase vulnerability through immunosuppression and frailty, it is independently associated with long-term mortality [42]. Without cause-specific mortality or competing risk analyses, the model cannot distinguish pneumonia-related risk from underlying oncologic prognosis [42,43]. Thus, its prognostic value reflects the severity of the hospital course rather than a baseline risk factor at presentation. Accordingly, the score should be viewed as a global measure of long-term vulnerability among 30-day CAP survivors, predicting all-cause rather than pneumonia-specific mortality.

The proposed score predicts 12-month all-cause mortality rather than CAP-specific mortality, as cause-specific death and competing risks could not be assessed. Some variables included in the model (such as corticosteroid use and hospital length of stay > 8 days) reflect in-hospital disease trajectory and clinical decision-making rather than baseline admission characteristics, which may limit the score’s applicability as a pure admission triage tool. Finally, although internal validation was performed using a randomly selected validation cohort from the study population, external validation in independent populations and healthcare settings is required before broad clinical implementation. 

## 5. Conclusions

The new score demonstrated weak-to-moderate discriminatory capacity for predicting post-acute all-cause mortality between 30 days and 12 months after hospital admission among patients with community-acquired pneumonia who survived the first 30 days. Although it showed higher discrimination than PSI and CURB-65 in the internal validation cohort, its modest overall performance and low positive predictive value limit its use as a standalone tool for identifying patients who will experience long-term mortality. Further external validation and assessment of clinical utility are warranted before its implementation in routine practice. 

## Figures and Tables

**Table 1:** General population characteristics.

	Derivation Cohort				Validation Cohort			
Variable	Total n = 1856	Alive n = 1556	Dead n = 300	*p* Value	Total n = 1832	Alive n = 1530	Dead n = 302	*p* Value
Age years, m (SD)	63.2 (21.4)	62 (21.78)	69.5 (18.08)	<0.05	63.7 (21.38)	62 (21.79)	71.9 (16.91)	<0.05
Male, n (%)	1100 (59.3)	913 (58.7)	187 (62.3)	0.238	1088 (59.4)	888 (58)	200 (66.2)	0.008
Sign and symptoms								
Cough, n (%)	1545 (83.3)	1319 (84.8)	226 (75.3)	<0.05	1500 (81.9)	1267 (82.8)	233 (77.2)	<0.05
Dyspnea, n (%)	1280 (69)	1095 (70.4)	185 (61.7)	<0.05	1206 (65.8)	1000 (65.4)	206 (68.2)	0.340
Fever, n (%)	888 (47.9)	765 (49.2)	123 (41)	<0.05	868 (47.4)	735 (48.1)	133 (44)	<0.05
Pleuritic pain, n (%)	464 (25)	415 (26.7)	49 (16.3)	<0.05	490 (26.7)	428 (28)	62 (20.5)	0.008
Cyanosis, n (%)	127 (6.8)	105 (6.8)	22 (7.3)	0.715	107 (5.8)	85 (5.6)	22 (7.3)	0.242
Chest retractions, n (%)	365 (19.7)	308 (19.8)	57 (19)	0.748	366 (20)	297 (19.4)	69 (22.8)	0.172
Altered consciousness, n (%)	174 (9.4)	120 (7.7)	54 (18)	<0.05	197 (10.8)	141 (9.2)	56 (18.5)	<0.05
Crackles, n (%)	954 (51.4)	820 (52.7)	134 (44.7)	<0.05	970 (52.9)	808 (52.8)	162 (53.6)	0.791
Wheezing, n (%)	420 (22.6)	369 (23.7)	51 (17)	<0.05	409 (22.3)	350 (22.9)	59 (19.5)	0.203
Heart rate bpm, m (SD)	91.5 (18.46)	91.5 (18.31)	91.1 (19.26)	0.711	91.2 (18.44)	91.5 (18.29)	89.6 (19.15)	0.120
Systolic BP mmHg, m (SD)	123.3 (21.68)	123.7 (21.04)	121 (24.76)	0.078	122 (21.28)	122.4 (20.32)	120.2 (25.75)	0.155
Diastolic BP mmHg, m (SD)	73.3 (12.69)	73.4 (12.35)	72.5 (14.37)	0.307	72.5 (13.57)	72.9 (13.09)	70.9 (15.81)	<0.05
Respiratory rate bpm, m (SD)	21.3 (4.8)	21.2 (4.7)	21.8 (5.3)	0.056	21.4 (4.73)	21.2 (4.6)	22.2 (5.25)	<0.05
Temperature °C, m (SD)	36.9 (0.91)	36.9 (0.93)	36.8 (0.77)	<0.05	36.9 (0.93)	36.9 (0.94)	36.7 (0.81)	<0.05
FiO_2_ admission %, m (SD)	28.6 (12.75)	28.4 (12.4)	29.3 (14.22)	0.340	28.4 (11.89)	27.7 (10.67)	31.2 (15.66)	<0.05
Comorbidities, n (%)								
Hypertension	839 (45.3)	690 (44.4)	149 (49.8)	0.082	860 (47)	696 (45.5)	164 (54.3)	0.005
Smoking	336 (18.1)	281 (18.1)	55 (18.4)	0.894	332 (18.1)	266 (17.4)	66 (21.9)	0.066
Chronic heart failure	217 (11.7)	176 (11.3)	41 (13.7)	0.238	230 (12.6)	181 (11.8)	49 (16.2)	<0.05
AMI	89 (4.8)	72 (4.6)	17 (5.7)	<0.05	79 (4.3)	64 (4.2)	15 (5)	0.542
PVD	59 (3.2)	49 (3.2)	10 (3.3)	0.862	51 (2.8)	39 (2.6)	12 (4)	0.170
Stroke	245 (7.1)	125 (6.1)	120 (12)	<0.05	239 (6.9)	126 (6.4)	113 (9.6)	<0.05
Chronic lung disease	472 (25.5)	381 (24.5)	91 (30.4)	<0.05	469 (25.6)	389 (25.4)	80 (26.5)	0.703
Liver disease	12 (0.6)	8 (0.5)	4 (1.3)	0.104	10 (0.5)	8 (0.5)	2 (0.7)	0.764
Diabetes Mellitus	42 (2.3)	36 (2.3)	6 (2)	0.743	62 (3.4)	47 (3.1)	15 (5)	0.097
CKD	94 (5.1)	73 (4.7)	21 (7)	0.093	107 (5.8)	76 (5)	31 (10.3)	<0.05
Tumor	123 (6.6)	91 (5.9)	32 (10.7)	<0.05	114 (6.2)	77 (5)	37 (12.3)	<0.05
Metastases	35 (1.9)	22 (1.4)	13 (4.3)	<0.05	27 (1.5)	18 (1.3)	12 (4)	0.099

n: number; m: mean; SD: standard deviation; ACM: Acute myocardial infarction; PVD: Peripheral vascular disease; CKD: chronic kidney disease.

**Table 2:** Cox regression.

Variable	Derivation Cohort			Validation Cohort		
	HR	95% CI	*p* Value	HR	95% CI	*p* Value
Altered mental status	2.077	1.487–2.901	<0.05	1.650	1.200–2.269	<0.05
Multilobar/bilateral infiltrate on chest X-ray	1.459	1.104–1.927	<0.05	1.527	1.161–2.009	<0.05
BUN > 30	1.975	1.482–2.631	<0.05	1.373	1.032–1.828	<0.05
Temperature < 35 or >39.9 °C	1.978	1.429–2.737	<0.05	1.350	0.632–2.886	0.439
Age ≥ 70	1.223	0.934–1.601	0.143	1.592	1.208–2.096	<0.05
Neoplasm	1.582	1.080–2.318	<0.05	2.343	1.635–3.355	<0.05
CKD	1.203	0.727–1.990	0.472	1.695	1.134–2.532	<0.05
Hospital stay >8 days	1.390	1.062–1.819	<0.05	1.506	1.154–1.966	<0.05
Corticosteroid use	1.848	1.397–2.443	<0.05	1.570	1.192–2.068	<0.05

Notes: HR: hazard ratio; BUN: Blood urea nitrogen; CKD: chronic kidney disease.

**Table 3:** Performance of Risk Scores in Community-Acquired Pneumonia for 12 months Mortality Prediction.

Score	Se (CI 95%)	Sp (CI 95%)	PPV (CI 95%)	NPV (CI 95%)	LR+ (CI 95%)	LR− (CI 95%)	ROC-Curve (CI 95%)	*p* Value *
CURB-65	65.9 (63.2–68.5)	53.2 (50.2–56)	22.5 (20.1–24.8)	88.3 (86.5–90.1)	1.41 (1.21–1.63)	0.64 (0.55–0.74)	0.63 (0.59–0.67)	<0.05
CRB-65	40.9 (38.5–43.3)	81.3 (80.1–83.2)	29.3 (27.1–31.5)	87.9 (86.3–89.5)	2.19 (1.68–2.84)	0.73 (0.55–0.94)	0.63 (0.59–0.67)	<0.05
SCAP	19.5 (3.2–43.1)	88.6 (86.4–90.7)	28.2 (25.2–31.2)	82.7 (80.1–85.2)	1.7 (1.07–2.69)	0.91 (0.57–1.43)	0.59 (0.55–0.65)	<0.05
CORB	29 (26.8–31.3)	80.9 (78.9–82.8)	23 (20.9–25.1)	85.3 (83.5–87)	1.52 (1.16–1.97)	0.88 (0.67–1.14)	0.57 (0.53–0.61)	<0.05
ADROP	41.5 (38.7–44.3)	75.8 (73.4–78.2)	26.6 (24.1–29.1)	86 (84.1–88)	1.72 (1.33–2.20)	0.77 (0.601–0.99)	0.62 (0.59–0.66)	<0.05
NEWS	47.3 (44.6–50.1)	63.8 (61.1–66.4)	20 (17.8–22.2)	86.3 (84.4–88.2)	1.31 (1.07–1.58)	0.83 (0.68–1.01)	0.59 (0.55–0.63)	<0.05
Pneumonia shock	65.4 (62.4–68.5)	60.9 (50.1–64)	29.4 (26.5–32.3)	87.6 (85.5–89.7)	1.67 (1.38–2.01)	0.57 (0.47–0.68)	0.64 (0.6–0.69)	<0.05
REA ICU	32.4 (29–35.9)	77.8 (74.7–80.9)	27.5 (24.2–30.8)	81.6 (78.8–84.5)	1.46 (1.05–2.02)	0.87 (0.62–1.20)	0.60 (0.55–0.65)	<0.05
PSI	85.3 (49.8–154.7)	27.8 (25.7–29.9)	18.2 (16.3–20)	91 (89.6–92.3)	1.18 (1.13–1.22)	0.53 (0.51–0.54)	0.65 (0.62–0.69)	<0.05
SMART-COP	59.9 (57.7–67)	51.2 (48.9–53.5)	19.5 (17.7–21.3)	86.6 (85.1–88.2)	1.23 (1.08–1.38)	0.78 (0.69–0.88)	0.52 (0.37–0.68)	0.776
SMRT-CO	52.3 (49.9–54.7)	67.3 (65.1–69.6)	23.1 (21.1–25.2)	88.3 (86.7–89.8)	1.6 (1.33–1.92)	0.71 (0.59–0.85)	0.60 (0.57–0.64)	<0.05
SOAR	54.8 (51.6–58)	53.8 (50.7–57)	21.2 (18.6–23.8)	84 (81.7–86.3)	1.19 (1–1.40)	0.84 (0.70–0.99)	0.56 (0.51–0.61)	<0.05
qSOFA	15.9 (14.2–17.6)	90.3 (89–91.7)	24.5 (22.5–26.5)	84.5 (82.8–86.1)	1.64 (1.14–2.36)	0.93 (0.64–1.33)	0.58 (0.54–0.62)	<0.05
SIRS	56.3 (54–58.6)	43 (40.7–45.3)	16.3 (14.6–18)	83.3 (81.6–85)	0.99 (0.88–1.1)	1.02 (0.93–1.13)	0.48 (0.45–0.52)	0.392
CAPSI	17.8 (15.7–20)	91.4 (89.8–92.9)	29.9 (27.4–32.5)	84.3 (82.3–86.3)	2.06 (1.30–3.25)	1.11 (0.70–1.75)	0.62 (0.58–0.67)	<0.05
Charlson	75.5 (73.5–77.5)	51.7 (49.4–54)	23.6 (21.6–25.5)	91.4 (90.2–92.7)	1.56 (1.38–1.76)	0.47 (0.42–0.53)	0.65 (0.62–0.68)	<0.05
New score	83 (81.3–84.7)	50.9 (48.6–53.2)	8.9 (7.6–10.2)	99 (98.5–99.5)	1.69 (1.38–2.06)	0.33 (0.27–0.40)	0.69 (0.66–0.73)	<0.05

Notes: Se: sensitivity; Sp: specificity; PPV: positive predictive value; NPV: negative predictive value; LR: likelihood ratio; ROC-curve: Area under the receiver operating characteristic curve. * Comparison of ROC curves utilizing the DeLong test.

## Data Availability

The de-identified datasets generated during and/or analyzed during the current study are available from the corresponding author on reasonable request.

## Supplementary Materials

The following supporting information can be downloaded at: https://www.mdpi.com/article/10.3390/idr18050103/s1, Table S1: Risk scores used in the study, Table S2: Cohort points in risk scores, Table S3: Proportion of missing data for candidate predictors in the derivation and validation cohorts, Table S4: laboratory tests and diagnostic imaging, Table S5: Assessment of the proportional hazards assumption using scaled Schoenfeld residuals, Table S6: Calibration performance of the prognostic score in the validation cohort.
